# Supplementary material for: Whole Genome Sequencing Differentiates Presumptive Extended Spectrum Beta-Lactamase Producing Escherichia coli along Segments of the One Health Continuum
Source: Microorganisms. 2020 Mar 22;8(3):448. doi: 10.3390/microorganisms8030448 (PMC7143971; doi:10.3390/microorganisms8030448)
Supplement: Supplementary file 1 [file microorganisms-08-00448-s001.zip › Adator_Fig_S1_to_S6_Table_S4_S5_S57_S12_supplementary file_clean_revised.docx]

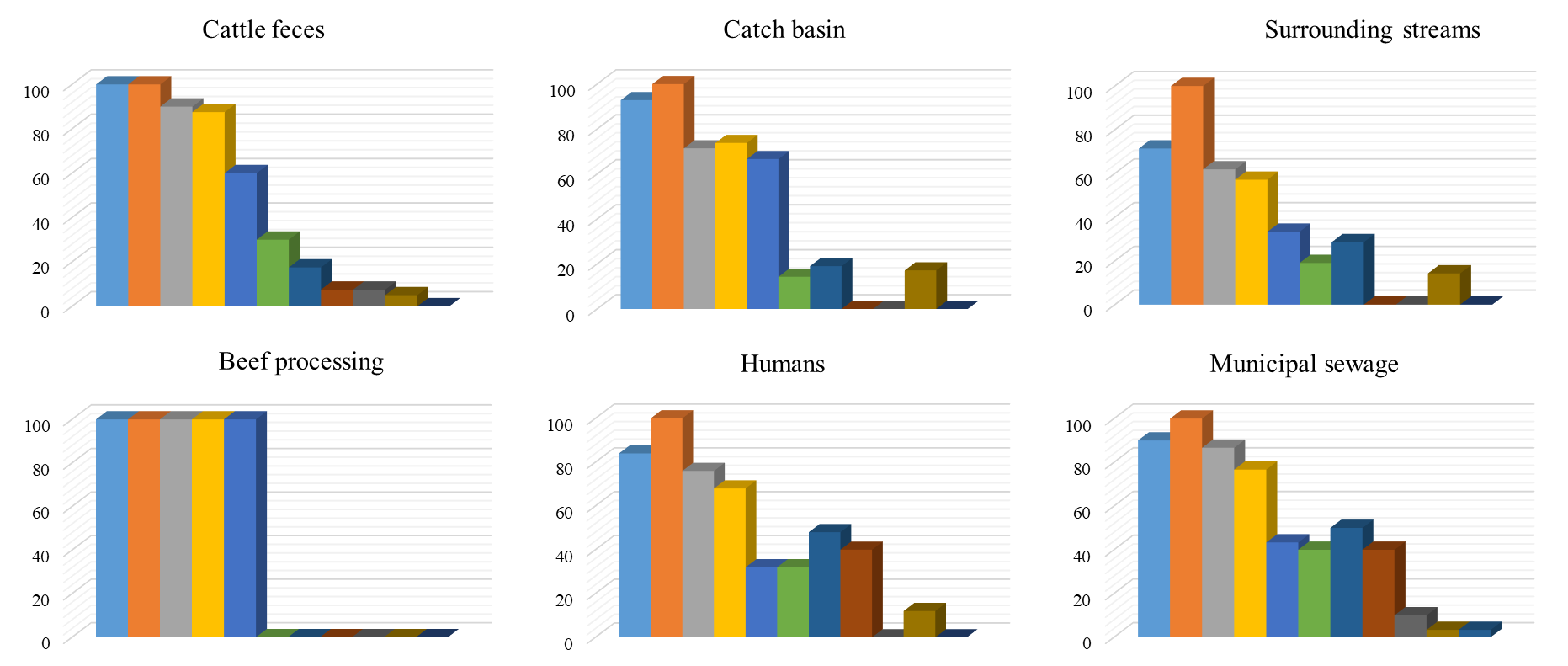

**Figure S1**: Occurrence (%) of genes that encode resistance for antibiotics in whole genome sequences of presumptive ESBL *E. coli* per source of isolates.

**Table S5**: Least square means comparing antimicrobial resistance gene prevalence in cattle versus human isolates

| Antimicrobial resistance *gene | Human | Cattle | P value |
| --- | --- | --- | --- |
| *sul1* | 0.4^a^ | 0.18^b^ | 0.007 |
| *sul2* | 0.53^b^ | 0.72^a^ | 0.024 |
| *blaEC* | 0.04^b^ | 0.249^a^ | 0.006 |
| *blaEC-8* | 0.24^a^ | 0.05^b^ | 0.003 |
| *blaEC-15* | 0.073^b^ | 0.22^a^ | 0.030 |
| *blaEC-18* | 0.055^b^ | 0.45^a^ | <.0001 |
| *blaTEM-1* | 0.36^a^ | 0.18^b^ | 0.021 |
| *blaCTX-M-15* | 0.55^a^ | 0.2^b^ | <.0001 |
| *blaCTX-M-55* | 0.091^b^ | 0.27^a^ | 0.016 |
| *blaCMY-2* | 0.036^b^ | 0.3^a^ | 0.002 |
| *aph(3'')-Ib* | 0.51^b^ | 0.73^a^ | 0.01 |
| *aph(6)-Id* | 0.49^b^ | 0.71^a^ | 0.013 |
| *floR* | 0.49 | 0.71^a^ | <.0001 |
| *mph(A)* | 0.38^a^ | 0.04^b^ | <.0001 |
| *qacEdelta1* | 0.42^a^ | 0.18^b^ | 0.004 |

Among the antimicrobial resistance genes associated with resistance phenotypes 13 genes for which *significant difference were observed are presented. a,b. least squares means differ by P < 0.05.

**Figure S2**: Prevalence of genetic determinants of antimicrobial resistance in human- versus cattle-sourced *E. coli* isolates. (i) Humans comprise all isolates from municipal sewage and clinically ill patients, while (ii) Cattle comprise all isolates from cattle feces, catch basins, surface streams and beef processing plant. Determinants included β-lactams (*bla* genes), tetracyclines (*tet* genes), aminoglycoside (*aac, aad, aph*, *armA and sat* genes)*,* folate synthesis inhibitors (*sul* and *dfrA* genes), phenicol (*catB, cmlA, cmlA* and *floR* genes), quinolone (*qnrS, qnrB* and *aac(6')Ib-cr* genes) and quaternary ammonium compound (*qacG, qacEdelta* and *qacL* genes).

**Table S6**: Genotype and phenotype comparison of presumptive ESBL *E. coli* isolates from multiple sources of the One Health continuum

|  | Phenotype: resistant | |  | Phenotype: susceptible | |  |  |  |  |  |
| --- | --- | --- | --- | --- | --- | --- | --- | --- | --- | --- |
| **Antimicrobial agent** | **Gene positive** | **Gene negative** |  | **Gene positive** | **Gene negative** | Sensitivity (%) | Specificity (%) | PPV (%) | NPV (%) |  |
| **Aminoglycoside** |  |  |  |  |  |  |  |  |  |  |
| ***Streptomycin*** |  |  |  |  |  |  |  |  |  |  |
| *All isolates total* | 112.00 | 3.00 |  | 16.00 | 31.00 | 97.39 | 65.96 | 78.32 | 91.20 |  |
| Cattle feces | 35.00 | 0.00 |  | 1.00 | 4.00 | 100.00 | 80.00 | 89.74 | 100.00 |  |
| Catch Basin | 30.00 | 2.00 |  | 0.00 | 10.00 | 93.75 | 100.00 | 75.00 | 83.30 |  |
| Surface water | 13.00 | 1.00 |  | 0.00 | 7.00 | 92.86 | 100.00 | 65.00 | 87.50 |  |
| Processing plant | 3.00 | 0.00 |  | 1.00 | 0.00 | 100.00 | 0.00 | 100.00 | - |  |
| *Cattle total* | 81.00 | 3.00 |  | 2.00 | 21.00 | 96.43 | 91.30 | 79.41 | 87.50 |  |
| Sewage treatment | 20.00 | 0.00 |  | 6.00 | 4.00 | 100.00 | 40.00 | 76.92 | 100. 00 |  |
| Humans | 11.00 | 0.00 |  | 8.00 | 6.00 | 100.00 | 42.86 | 57.89 | 100. 00 |  |
| *Human total* | 31.00 | 0.00 |  | 14.00 | 10.00 | 100.00 | 41.67 | 68.89 | 100. 00 |  |
|  |  |  |  |  |  |  |  |  |  |  |
| ***Neomycin*** |  |  |  |  |  |  |  |  |  |  |
| *All isolates total* | 8.00 | 0.00 |  | 96.00 | 58.00 | 100.00 | 37.66 | 12.12 | 100.00 |  |
| Cattle feces | 3.00 | 0.00 |  | 31.00 | 6.00 | 100.00 | 16.22 | 33.33 | 100.00 |  |
| Catch Basin | 1.00 | 0.00 |  | 28.00 | 13.00 | 100.00 | 31.71 | 7.14 | 100.00 |  |
| Surface water | 0.00 | 0.00 |  | 12.00 | 9.00 | - | 42.86 | 0.00 | 100.00 |  |
| Processing plant | 0.00 | 0.00 |  | 0.00 | 4.00 | - | 100.00 | 0.00 | 100.00 |  |
| *Cattle total* | 4.00 | 0.00 |  | 71.00 | 32.00 | 100.00 | 31.07 | 11.11 | 100.00 |  |
| Sewage treatment | 3.00 | 0.00 |  | 17.00 | 10.00 | 100.00 | 37.04 | 15.00 | 100.00 |  |
| Humans | 1.00 | 0.00 |  | 8.00 | 16.00 | 100.00 | 66.67 | 11.11 | 100.00 |  |
| *Human total* | 4.00 | 0.00 |  | 25.00 | 26.00 | 100.00 | 50.98 | 13.79 | 100.00 |  |
|  |  |  |  |  |  |  |  |  |  |  |
| **beta-lactam/beta-lactam inhibitor** | |  |  |  |  |  |  |  |  |  |
| *Cephems* |  |  |  |  |  |  |  |  |  |  |
| ***Ceftazidime*** |  |  |  |  |  |  |  |  |  |  |
| *All isolates total* | 63.0 | 61.0 |  | 74.0 | 25.0 | 96.8 | 25.30 | 46.0 | 29.10 |  |
| Cattle feces | 16.0 | 15.0 |  | 19.0 | 5.0 | 93.8 | 20.80 | 45.7 | 25.00 |  |
| Catch Basin | 22.0 | 21.0 |  | 11.0 | 9.0 | 95.5 | 45.00 | 66.7 | 30.00 |  |
| Surface water | 5.0 | 5.0 |  | 5.0 | 11.0 | 100.0 | 68.80 | 50.0 | 68.80 |  |
| Processing plant | 3.0 | 3.0 |  | 1.0 | 0.0 | 100.0 | 0.00 | 75.0 | - |  |
| *Cattle total* | 46.0 | 44.0 |  | 36.0 | 25.0 | 95.7 | 41.00 | 56.1 | 36.20 |  |
| Sewage treatment | 12.0 | 0.0 |  | 18.0 | 0.0 | 40.0 | 0.00 | 40.0 | - |  |
| Humans | 5.0 | 0.0 |  | 20.0 | 0.0 | 20.0 | 0.0 | 20.0 | - |  |
| *Human total* | 17.0 | 0.0 |  | 38.0 | 0.0 | 30.9 | 0.0 | 30.9 | - |  |
|  |  |  |  |  |  |  |  |  |  |  |
| ***Ceftiofor*** |  |  |  |  |  |  |  |  |  |  |
| *All isolates total* | 127.0 | 3.0 |  | 8.0 | 24.0 | 97.7 | 75.0 | 84.1 | 88.90 |  |
| Cattle feces | 33.0 | 2.0 |  | 1.0 | 4.0 | 94.3 | 80.0 | 89.2 | 66.70 |  |
| Catch Basin | 29.0 | 1.0 |  | 3.0 | 9.0 | 96.7 | 75.0 | 76.3 | 90.00 |  |
| Surface water | 10.0 | 0.0 |  | 0.0 | 11.0 | 100.0 | 100.0 | 47.6 | 100.00 |  |
| Processing plant | 4.0 | 0.0 |  | 0.0 | 0.0 | 100.0 | - | 100.0 | - |  |
| *Cattle total* | 76.0 | 3.0 |  | 4.0 | 24.0 | 96.2 | 85.7 | 76.0 | 88.90 |  |
| Sewage treatment | 28.0 | 0.0 |  | 2.0 | 0.0 | 100.0 | 0.0 | 93.3 | - |  |
| Humans | 23.0 | 0.0 |  | 2.0 | 0.0 | 100.0 | 0.0 | 92.0 | - |  |
| *Human total* | 51.0 | 0.0 |  | 4.0 | 0.0 | 100.0 | 0.0 | 92.7 | - |  |
|  |  |  |  |  |  |  |  |  |  |  |
| *Penicillin* |  |  |  |  |  |  |  |  |  |  |
| ***Ampicillin*** |  |  |  |  |  |  |  |  |  |  |
| *All isolates total* | 136.0 | 10.0 |  | 0.0 | 16.0 | 93.2 | 100.0 | 100.0 | 61.50 |  |
| Cattle feces | 35.0 | 4.0 |  | 0.0 | 1.0 | 89.7 | 100.0 | 100.0 | 20.00 |  |
| Catch Basin | 32.0 | 3.0 |  | 0.0 | 7.0 | 91.4 | 100.0 | 100.0 | 70.00 |  |
| Surface water | 10.0 | 3.0 |  | 0.0 | 8.0 | 76.9 | 100.0 | 100.0 | 72.73 |  |
| Processing plant | 4.0 | 0.0 |  | 0.0 | 0.0 | 100.0 | - | 100.0 | - |  |
| *Cattle total* | 81.0 | 10.0 |  | 0.0 | 16.0 | 89.0 | 100.0 | 100.0 | 61.50 |  |
| Sewage treatment | 30.0 | 0.0 |  | 0.0 | 0.0 | 100.0 | - | 100.0 | - |  |
| Humans | 25.0 | 0.0 |  | 0.0 | 0.0 | 100.0 | - | 100.0 | - |  |
| *Human total* | 55.0 | 0.0 |  | 0.0 | 0.0 | 100.0 | - | 100.0 | - |  |
|  |  |  |  |  |  |  |  |  |  |  |
| **Folate pathway inhibitors** |  |  |  |  |  |  |  |  |  |  |
| ***Trimethoprim/sulfamethoxazole*** |  |  |  |  |  |  |  |  |  |  |
| *All isolates total* | 93.0 | 0.0 |  | 29.0 | 40.0 | 100.0 | 58.0 | 69.9 | 100.00 |  |
| Cattle feces | 31.0 | 0.0 |  | 4.0 | 5.0 | 100.0 | 55.6 | 86.1 | 100.00 |  |
| Catch Basin | 23.0 | 0.0 |  | 7.0 | 11.0 | 100.0 | 61.1 | 67.6 | 100.00 |  |
| Surface water | 8.0 | 0.0 |  | 5.0 | 9.0 | 100.0 | 64.3 | 47.1 | 100.00 |  |
| Processing plant | 0.0 | 0.0 |  | 4.0 | 0.0 | - | 0.0 | - | - |  |
| *Cattle total* | 62.0 | 0.0 |  | 20.0 | 25.0 | 100.0 | 55.6 | 71.3 | 100.00 |  |
| Sewage treatment | 18.0 | 0.0 |  | 5.0 | 7.0 | 100.0 | 58.3 | 78.3 | 100.00 |  |
| Humans | 13.0 | 0.0 |  | 4.0 | 8.0 | 100.0 | 66.7 | 76.5 | 100.00 |  |
| *Human total* | 31.0 | 0.0 |  | 9.0 | 15.0 | 100.0 | 62.5 | 77.5 | 100.00 |  |
|  |  |  |  |  |  |  |  |  |  |  |
| ***Sulfisoxazole*** |  |  |  |  |  |  |  |  |  |  |
| *All isolates total* | 112.0 | 5.0 |  | 6.0 | 39.0 | 95.7 | 86.7 | 74.2 | 88.60 |  |
| Cattle feces | 34.0 | 0.0 |  | 0.0 | 6.0 | 100.0 | 100.0 | 85.0 | 100.00 |  |
| Catch Basin | 23.0 | 3.0 |  | 4.0 | 10.0 | 88.5 | 71.4 | 69.7 | 76.92 |  |
| Surface water | 14.0 | 2.0 |  | 0.0 | 7.0 | 87.5 | 100.0 | 66.7 | 77.80 |  |
| Processing plant | 3.0 | 0.0 |  | 1.0 | 0.0 | 100.0 | 0.0 | 100.0 | - |  |
| *Cattle total* | 74.0 | 5.0 |  | 5.0 | 23.0 | 93.7 | 82.1 | 76.3 | 82.14 |  |
| Sewage treatment | 22.0 | 0.0 |  | 0.0 | 8.0 | 100.0 | 100.0 | 100.0 | 100.00 |  |
| Humans | 16.0 | 0.0 |  | 1.0 | 8.0 | 100.0 | 88.9 | 94.1 | 100.00 |  |
| *Human total* | 38.0 | 0.0 |  | 1.0 | 16.0 | 100.0 | 94.1 | 97.4 | 100.00 |  |
|  |  |  |  |  |  |  |  |  |  |  |
| **Phenicol** |  |  |  |  |  |  |  |  |  |  |
| ***Florfenicol*** |  |  |  |  |  |  |  |  |  |  |
| *All isolates total* | 67.0 | 3.0 |  | 17.0 | 75.0 | 95.7 | 81.5 | 47.2 | 96.20 |  |
| Cattle feces | 23.0 | 2.0 |  | 1.0 | 14.0 | 92.0 | 93.3 | 62.2 | 87.50 |  |
| Catch Basin | 28.0 | 1.0 |  | 0.0 | 13.0 | 96.6 | 100.0 | 68.3 | 92.90 |  |
| Surface water | 7.0 | 0.0 |  | 0.0 | 14.0 | 100.0 | 100.0 | 33.3 | 100.0 |  |
| Processing plant | 3.0 | 0.0 |  | 1.0 | 0.0 | 100.0 | 0.0 | 100.0 | - |  |
| *Cattle total* | 61.0 | 3.0 |  | 2.0 | 41.0 | 95.3 | 95.3 | 59.8 | 93.20 |  |
| Sewage treatment | 5.0 | 0.0 |  | 8.0 | 17.0 | 100.0 | 68.0 | 38.5 | 100.00 |  |
| Humans | 1.0 | 0.0 |  | 7.0 | 17.0 | 100.0 | 70.8 | 12.5 | 100.00 |  |
| *Human total* | 6.0 | 0.0 |  | 15.0 | 34.0 | 100.0 | 71.4 | 28.6 | 100.00 |  |
|  |  |  |  |  |  |  |  |  |  |  |
| **Quinolones** |  |  |  |  |  |  |  |  |  |  |
| ***Enrofloxacin*** |  |  |  |  |  |  |  |  |  |  |
| *All isolates total* | 70.0 | 0.0 |  | 91.0 | 1.0 | 100.0 | 1.1 | 43.5 | 100.00 |  |
| Cattle feces | 19.0 | 0.0 |  | 21.0 | 0.0 | 100.0 | 0.0 | 47.5 | - |  |
| Catch Basin | 8.0 | 0.0 |  | 27.0 | 0.0 | 100.0 | 0.0 | 22.9 | - |  |
| Surface water | 2.0 | 0.0 |  | 25.0 | 1.0 | 100.0 | 3.8 | 7.4 | 100.00 |  |
| Processing plant | 0.0 | 0.0 |  | 4.0 | 0.0 | 0.0 | 0.0 | 0.0 | - |  |
| *Cattle total* | 29.0 | 0.0 |  | 77.0 | 1.0 | 100.0 | 1.3 | 27.4 | 100.00 |  |
| Sewage treatment | 23.0 | 0.0 |  | 7.0 | 0.0 | 100.0 | 0.0 | 76.7 | - |  |
| Humans | 18.0 | 0.0 |  | 7.0 | 0.0 | 100.0 | 0.0 | 72.0 | - |  |
| *Human total* | 41.0 | 0.0 |  | 14.0 | 0.0 | 100.0 | 0.0 | 74.5 | - |  |
|  |  |  |  |  |  |  |  |  |  |  |
| **Tetracyclines** |  |  |  |  |  |  |  |  |  |  |
| ***Oxytertracycline*** |  |  |  |  |  |  |  |  |  |  |
| *All isolates total* | 144.0 | 6.0 |  | 2.0 | 11.0 | 96.6 | 84.6 | 98.6 | 64.70 |  |
| Cattle feces | 40.0 | 0.0 |  | 0.0 | 0.0 | 100.0 | - | 100.0 | - |  |
| Catch Basin | 39.0 | 3.0 |  | 0.0 | 1.0 | 92.9 | 100.0 | 100.0 | 25.00 |  |
| Surface water | 15.0 | 2.0 |  | 0.0 | 4.0 | 88.2 | 100.0 | 100.0 | 66.70 |  |
| Processing plant | 3.0 | 0.0 |  | 1.0 | 0.0 | 100.0 | 0.0 | 75.0 | - |  |
| *Cattle total* | 97.0 | 5.0 |  | 1.0 | 5.0 | 95.1 | 83.3 | 99.0 | 50.00 |  |
| Sewage treatment | 27.0 | 0.0 |  | 0.0 | 3.0 | 100.0 | 100.0 | 100.0 | 100.00 |  |
| Humans | 20.0 | 1.0 |  | 1.0 | 3.0 | 100.0 | 75.0 | 95.2 | 75.00 |  |
| *Human total* | 47.0 | 1.0 |  | 1.0 | 6.0 | 100.0 | 85.7 | 97.9 | 85.70 |  |
| Sensitivity (%) equals number of isolates that harbored resistant determinants divided by the total number of isolates exhibiting clinical resistance phenotypes, while specificity (%) was calculated as the number of isolates that did not harbor genetic determinants divided by the total number of phenotypically susceptible isolates. Whole genome sequence positive predictive value (PPV%) and negative predictive value (NPV%). | | | | | | | | | |  |
| Genes considered for encoding resistance to antimicrobial included:  (I) **Streptomycin**: *aph(3')-IIa, aph(3')-Ia, aph(3'')-Ib, aph(6)-Ic, aph(6)-Id, aac(3)-IIa, aac(3)-IId, aac(3)-Via, aadA1, aadA2, aadA5, aadA16, aadA22,* *StrA*  (II) **Neomycin:** aph(3')-Ia, aph(3'')-Ib, aph(6)-Ic, aph(6)-Id  (III) **Ceftazidime:** bla_TEM-1_,bla_TEM-15_, bla_TEM-104_, bla_TEM-150_, bla_CTX-M-1_, bla_CTX-M-14_, bla_CTX-M-15_, bla_CTX-M-27_, bla_CTX-M-55_, bla_CMY-42_, bla_CTX-M-65_, bla_CMY-2_  (IV) **Ceftiofor:** bla_TEM-1_,bla_TEM-15_, bla_TEM-104_, bla_TEM-150_, bla_CTX-M-1_, bla_CTX-M-14_, bla_CTX-M-15_, bla_CTX-M-27_, bla_CTX-M-55_, bla_CMY-42_, bla_CTX_-_M-65_, bla_CMY-2_  (V) **Ampicillin**: bla_LAP-2_, bla_TEM-1_, bla_TEM-15_,bla_TEM-104_, bla_TEM-150_, bla_CTX-M-1_, bla_CTX-M-14_, bla_CTX-M-15_, bla_CTX-M-27_, bla_CTX-M-55_, bla_CTX-M-65_, bla_CMY-42_, bla_CMY-2_, bla_OXA-1_, bla_OXA-10_, bla_SHV-11_, bla_SHV-12_  (VII) **Trimethoprim/sulfamethoxazole:** *sul1, sul2, sul3, dfrA1, dfrA7, dfrA17, dfrA12, dfrA14, dfrA18, dfrA19, dfrA27*  (VIII) **Sulfisoxazole:** *sul1, sul2, sul3*  (IX) **Florfenicol:** *floR, catA1, catA2, catB3, catB4, cmlA1, cmlA5*  (X) **Enrofloxacin:** *qnrS1, qnrB2, QRDR Mutations of GyrA, GyrB, ParC, ParE*  (XI) **Oxytertracycline:** *tet(A), tet(B), tet(C), tet(M), tet(32)* | | | | | | | | | |  |
|  | | | | | | | | | |  |
|  | | | | | | | | | |  |

**Figure S3**: (A) Pairwise SNP differences between clinical human isolates (n = 25) and municipal sewage isolates (n=30), processing plants (n = 4), surrounding streams (n=21), catch basins (n=42), cattle feces (n=40). (B) Pairwise SNP differences between municipal sewage isolates (*n* = 30) and clinical human isolates (n=25), processing plants (*n* = 4), surrounding streams (n=21), catch basins (n=42) and cattle feces (n=40). Frequency represents the number of (A) clinical human and (B) municipal sewage isolates related to isolates from the respective sources at threshold SNPs defined on the *x* axis; SNP distance shown range from 0 to 500.

**Figure S4:** Whole genome MLST of presumptive ESBL-producing *E. coli* genomes along the One Health continuum from human clinical, municipal sewage, beef processing, cattle feces, catch basins, and surface streams generated using 9580 wgMLST loci, colored based on isolate origin. Bracket ( ) indicate clusters that are within 10 wgMLST alleles.


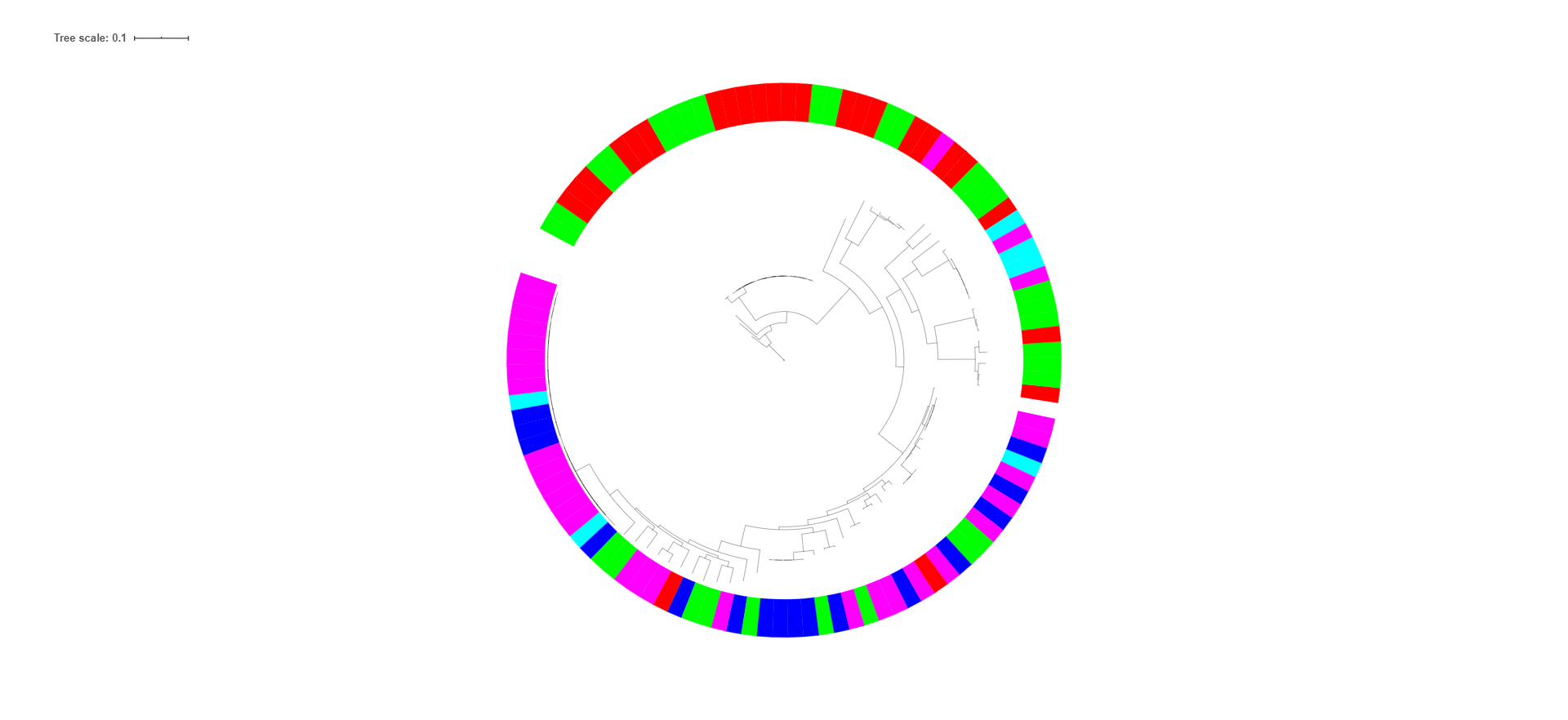

**Figure S5**: Phylogenetic tree generated based on single-nucleotide polymorphisms (SNPs) of the core genes of 108 *E. coli* isolates that proved to be true ESBL producers (*bla_TEM_, bla_CTX-M_, bla_OXA_* and *bla_SHV_*) obtained from cattle feces, catch basin, surface streams, municipal sewage and human clinical isolates from the One Health continuum, with reference genome *E. coli* str. K-12 substr. MG1655 (GenBank accession # GI: 545778205/ U00096.3). Beef processing plant isolates did not carry any ESBL gene.

**Figure S6**: Relative abundance of 38 plasmid types in ESBL-producing *E. coli* from the One Health continuum of the beef-production system.

**Table S8**: Differences between plasmid least square means estimates of the effect of isolate from cattle versus human sources

| *Plasmid | Human | Cattle | P value |
| --- | --- | --- | --- |
| Col(MG828) | 0.31^a^ | 0.073^b^ | 0.001 |
| ColRNAI | 0.39^a^ | 0.21^b^ | 0.0188 |
| Col156 | 0.4^a^ | 0.037^b^ | <.0001 |
| IncFIA | 0.56^a^ | 0.02^b^ | <.0001 |
| IncFIB(AP001918) | 0.76 ^a^ | 0.34^b^ | <.0001 |
| IncFII | 0.6^a^ | 0.40^b^ | 0.026 |
| IncFIC(FII) | 0.35^a^ | 0.098^b^ | 0.0009 |
| p0111 | 0.09 ^b^ | 0.27^a^ | 0.0157 |

Among the 38 plasmids, only plasmids for which *significant difference were observed are presented. a,b. least squares mean estimates differ by P < 0.05.

**Table S12**: Differences among virulence determinants identified in presumptive ESBL – *E. coli* isolated from different sources across a One Health continuum.

| Virulence gene | Human | Cattle | P value |
| --- | --- | --- | --- |
| *aslA* | 0.87^a^ | 0.39^b^ | <.0001 |
| *chuS* | 0.73^a^ | 0.04^b^ | <.0001 |
| *chuT* | 0.56^a^ | 0.01^b^ | <.0001 |
| *chuU* | 0.8^a^ | 0.05^b^ | <.0001 |
| *chuV* | 0.8^a^ | 0.05^b^ | <.0001 |
| *chuW* | 0.8^a^ | 0.05^b^ | <.0001 |
| *chuY* | 0.78^a^ | 0.04^b^ | <.0001 |
| *espL1* | 0.47^b^ | 0.99^a^ | <.0001 |
| *espR1* | 0.42^b^ | 0.91^a^ | <.0001 |
| *espX1* | 0.44^b^ | 0.82^a^ | <.0001 |
| *espX4* | 0.51^b^ | 0.88^a^ | <.0001 |
| *espX5* | 0.36^b^ | 0.98^a^ | <.0001 |
| *espY1* | 0.22^a^ | 0.09^b^ | 0.03 |
| *espY2* | 0.22^a^ | 0.02^b^ | 0.003 |
| *espY3* | 0.22^a^ | 0.04^b^ | 0.003 |
| *fimA* | 0.8^a^ | 0.56^b^ | 0.005 |
| *fyuA* | 0.68^a^ | 0.06^b^ | <.0001 |
| *gspL* | 0.91^a^ | 0.77^b^ | 0.04 |
| *hlyA* | 0.18^a^ | 0.04^b^ | 0.01 |
| *hlyB* | 0.18^a^ | 0.04^b^ | 0.01 |
| *hlyC* | 0.18^a^ | 0.05^b^ | 0.02 |
| *hlyD* | 0.18^a^ | 0.04^b^ | 0.01 |
| *irp1* | 0.67^a^ | 0.06^b^ | <.0001 |
| *irp2* | 0.67^a^ | 0.06^b^ | <.0001 |
| *iucA* | 0.65^a^ | 0.07^b^ | <.0001 |
| *iuB* | 0.65^a^ | 0.07^b^ | <.0001 |
| *iucC* | 0.65^a^ | 0.07^b^ | <.0001 |
| *iucD* | 0.65^a^ | 0.07^b^ | <.0001 |
| *kpsD* | 0.67^a^ | 0.01^b^ | <.0001 |
| *kpsM* | 0.62^a^ | 0.01^b^ | <.0001 |
| *papB* | 0.49^a^ | 0.012^b^ | <.0001 |
| *papC* | 0.25^a^ | 0.02^b^ | 0.001 |
| *papD* | 0.25^a^ | 0.02^b^ | 0.001 |
| *papF* | 0.25^a^ | 0.02^b^ | 0.001 |
| *papG* | 0.22^a^ | 0.02^b^ | 0.003 |
| *papH* | 0.25^a^ | 0.02^b^ | 0.001 |
| *papI* | 0.49^a^ | 0.02^b^ | <.0001 |
| *papJ* | 0.25^a^ | 0.02^b^ | 0.001 |
| *papK* | 0.25^a^ | 0.02^b^ | 0.001 |
| *papX* | 0.55^a^ | 0.02^b^ | <.0001 |
| *shuA* | 0.33^a^ | 0.05^b^ | 0.0002 |
| *shuT* | 0.24^a^ | 0.04^b^ | 0.002 |
| *shuX* | 0.29^a^ | 0.05^b^ | 0.0006 |
| *ybtA* | 0.67^a^ | 0.06^b^ | <.0001 |
| *ybtE* | 0.67^a^ | 0.06^b^ | <.0001 |
| *ybtP* | 0.67^a^ | 0.06^b^ | <.0001 |
| *ybtQ* | 0.67^a^ | 0.06^b^ | <.0001 |
| *ybtS* | 0.67^a^ | 0.06^b^ | <.0001 |
| *ybtT* | 0.67^a^ | 0.06^b^ | <.0001 |
| *ybtU* | 0.67^a^ | 0.06^b^ | <.0001 |
| *ybtX* | 0.67^a^ | 0.06^b^ | <.0001 |
